# Study protocol: pragmatic randomized control trial of an internet-based intervention (My tools 4 care) for family carers

**DOI:** 10.1186/s12877-017-0581-6

**Published:** 2017-08-14

**Authors:** Wendy Duggleby, Jenny Ploeg, Carrie McAiney, Kathryn Fisher, Jenny Swindle, Tracey Chambers, Sunita Ghosh, Shelley Peacock, Maureen Markle-Reid, Jean Triscott, Allison Williams, Dorothy Forbes, Lori Pollard

**Affiliations:** 1 Faculty of Nursing, University of Alberta, Level 3 Edmonton Clinic Health Academy, 11405 87 Avenue, Edmonton, AB T6G 1C9 Canada; 2 Aging, Community and Health Research Unit, School of Nursing, McMaster University, 1280 Main Street West, Room HSC3N25L, Hamilton, ON L8S 4K1 Canada; 3 Faculty of Health Sciences, McMaster University 1280 Main Street West, Room HSC3N25L, Hamilton, ON L8S 4K1 Canada; 4 Health Sciences, School of Nursing, McMaster University, 1280 Main Street West, Room HSC3N25L, Hamilton, ON L8S 4K1 Canada; 5 Department of Medical Oncology/Department of Mathematical and Statistical Sciences, University of Alberta, Edmonton, AB Canada; 6 College of Nursing, University of Saskatchewan, 4340 E-wing Health Sciences Building, 104 Clinic Place, Saskatoon, SK S7N 2Z4 Canada; 7 Department of Family Medicine & Director, Division of Care of the Elderly, Faculty of Medicine and Dentistry, University of Alberta, Edmonton, AB Canada; 8 School of Geography & Earth Sciences, McMaster University, Hamilton, ON Canada

**Keywords:** Carers, Dementia, Pragmatic Trial, Online intervention

## Abstract

**Background:**

Family carers of older persons with Alzheimer’s’ disease and related dementia (ADRD) and multiple chronic conditions (MCC) experience significant, complex, and distressing transitions such as changes to their environment, roles and relationships, physical health, and mental health. An online intervention (My Tools 4 Care) was developed for family carers of persons with ADRD and MCC living at home, with the aim of supporting these carers through transitions and increasing their self-efficacy, hope, and health related quality of life (HRQoL). This study will evaluate My Tools 4 Care (MT4C) by asking the following research questions:

1. Does use of MT4C result in a 3 month (immediately post intervention) and 6-month (3 months after intervention) increase in HRQoL, self-efficacy, and hope, in carers of persons with ADRD and MCC compared to an educational control group?
2. Does use of MT4C help carers of community-dwelling older adults with ADRD and MCC deal with significant changes they experience as carers? and
3. Are the effects/benefits of the MT4C intervention achieved at no additional cost compared to an educational control group?

**Methods/Design:**

Using a pragmatic mixed methods randomized controlled trial design, 180 family carers of community dwelling older persons (65 years of age and older) with ADRD and MCC will participate in the study. Data will be collected from the intervention and an educational control group at four time points: baseline, 1 month, 3 and 6 months. We expect to find that family carers using MT4C will show greater improvement in hope, self-efficacy and HRQoL, at no additional cost from a societal perspective, compared to those in the educational control group. General estimating equations will be used to determine differences between groups and over time.

**Discussion:**

Data collection began in Ontario and Alberta Canada in June 2015 and is expected to be completed in June 2017. The results will inform policy and practice as MT4C can be easily revised for local contexts and is scalable in terms of posting on websites such as those hosted by the Alzheimer Society.

**Trial registration:**

ClinicalTrials.gov Identifier: NCT02428387

## Background

With the escalating numbers of persons diagnosed with Alzheimer’s disease and related dementias (ADRD) world-wide [1], support for family carers of persons with dementia is critical because: a) they provide about 90% of in-home care for persons with ADRD [2], b) the care is often difficult and complex due to co-morbidities [multiple chronic conditions (MCC)] [3] and c) they experience significant, complex, and distressing transitions such as changes to their environment, roles and relationships, physical and mental health [4]. Adding to their distress is the lack of resources available for family carers including insufficient information to provide care [5]. Due to the nature of caring for someone with ADRD finding the time to access resources, even then they are available, is difficult [6, 7]. Online interventions show promise in supporting carers of persons with ADRD [8–10] as they provide flexibility for carers to access resources at a time and place that is convenient for them [6, 7, 11].

Three systematic reviews have evaluated studies of online-interventions developed for family carers of persons with ADRD [8–10]. Efficacious interventions with statistically significant results in caregiver health outcomes, were those that incorporated a personalized approach of choice to different parts of the intervention and were adaptable to an ever-changing set of needs and issues [6, 7, 12]. Interventions with the greatest impact offered multiple components [11–13] and were psycho-educational in nature. Pagan-Ortiz et al. [14] addressed the difficulty of ensuring that carers continue to return to their website by encouraging user-generated content, which ensured continued use of the website. However, none of the reviewed studies included a cost analysis of the intervention.

We developed a paper based psychosocial supportive intervention entitled “Transition Toolkit” for family carers of older persons with ADRD who are living at home. The purpose of the intervention, which is based on transition theory [15], was to support carers through transitions and increase their self-efficacy, hope, and health related quality of life (HRQoL). The intervention had multiple components and included choice, as the carers could choose which sections they would like use, and when. User-generated content is encouraged throughout the intervention as participants may write in sections, add stories, pictures, music etc. Sections include: a) About Me, b) Common Changes to Expect, c) Frequently Asked Questions, Resources, and d) Important Health Information (about person they are caring for). Data from a pilot study of the Transitions Toolkit suggest it is feasible, acceptable, and may support carers through transitions [16]. An online version of the Toolkit entitled My Tools 4 Care (MT4C) (https://www.mytools4care.ca/) was subsequently developed to improve its accessibility and portability.

The purpose of this paper is to describe the protocol for a pragmatic, mixed methods, multisite randomized control trial study designed to evaluate the effectiveness of the online MT4C intervention. The purpose of MT4C is to support family carers of community-living older persons with ADRD and MCC as they experience multiple transitions and to increase their HRQoL. The description follows the SPIRIT guidelines [17] which provides a list of the recommended items to include in clinical trial protocols.

### Study purpose

The purpose of the study is to evaluate the effectiveness of MT4C compared to an educational control group with respect to increasing hope, general self-efficacy and HRQoL and supporting participants through their experience of transitions over time. We expect to find that family carers who are allocated to MT4C intervention group will show greater improvement in hope, self-efficacy and HRQOL, at no additional cost from a societal perspective, compared to an educational control group. MT4C will be used for 3 months and we will be looking to see if the effects continue on after use for 6 months. The study will specifically address the following research questions:

1. Does use of MT4C result in a 3 month (immediately post intervention) and 6-month (3 months after intervention) increase in HRQoL, self-efficacy, and hope, in carers of persons with ADRD and MCC compared to an educational control group?
2. Does use of MyT4C help carers of community-dwelling older adults with ADRD and MCC deal with significant changes they experience as carers? and
3. Are the effects/benefits of the MT4C intervention achieved at no additional cost compared to an educational control group?

## Methods/Design

### Study design and participants

**Fig. 1:**
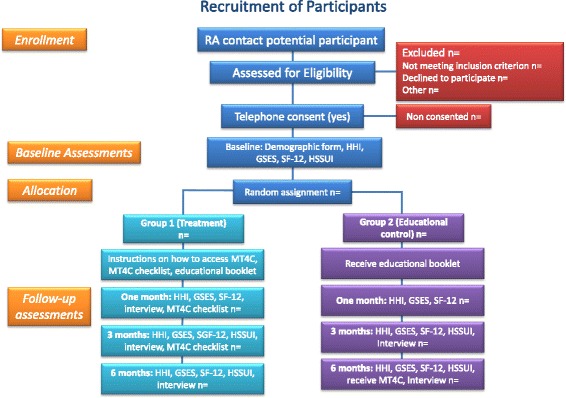
Study protocol MT4C

This is a multi-site, pragmatic, mixed methods, longitudinal, repeated measures, randomized controlled trial with randomized allocation to an intervention or educational control group. Recruitment and data collection began in June 2015 and is projected to be completed by June 2017. Participant data will be collected at intake (baseline) and at 1 month, 3 months, and 6 months from the beginning of the intervention. Cost data will be collected at baseline, then at 3 months and 6 months, as the cost measurement instrument asks participants to reflect upon the previous 3 months. Qualitative and quantitative data will be collected concurrently at select time points. The qualitative data will inform the quantitative data and will be integrated in the findings phase of the study. The study features two sites, one in Alberta and the other in Ontario. The CONSORT (Consolidated Standards of Reporting Trials) statement for non-pharmacological and pragmatic interventions was used to design the study and will be used to report study findings (Fig. 1). This study received ethical approval from the University of Alberta Health Research Ethics Board (# Pro00048721) and the Hamilton Integrated Research Ethics Board (#15–309).

### Inclusion and exclusion criteria

The study involves unpaid, family (broadly defined as family and friends) carers of older adults with ADRD and MCC, who are living at home. Participants will be considered eligible to participate in the study if they meet the following criteria: 1) carers of persons ≥65 years of age who have ADRD and two or more chronic conditions; 2) English-speaking, 3) family or friends ≥18 years of age who provide physical, emotional, or financial care to persons with ADRD and MCC; and 4) have an email address and access to a computer with internet. Potential participants are excluded if they are caring for a person with ADRD and MCC who: 1) has died, or 2) resides in a long-term care facility, or 3) who is hospitalized and designated as restorative care (Alberta) or alternate level of care (Ontario).

### Recruitment and randomization

Participants will be recruited through a variety of strategies. For example, advertisements in local community newspapers direct potential participants to contact the research coordinators (one in each province) via email or toll free number. The Alzheimer Societies (AS) of Brant, Haldimand Norfolk and Hamilton Halton in Ontario, and Alberta/NWT and Calgary in Alberta will also assist with recruiting potential participants by distributing study information (i.e., brochures, posters, and postcards) to interested carers. In Ontario a trained recruiter and in Alberta trained research assistants will attend local AS education and support groups and other community-based caregiver information sessions and support groups to share information about the study to potential participants.

AS staff members in each province will identify potential participants who may be interested in learning about the study. With their consent, potential participants will be contacted by a research coordinator (Ontario) or research assistants (Alberta). Potential participants will be contacted to inform them about the study and screen them for eligibility to participate. Names and contact information of carers who meet the study’s eligibility criteria will be forwarded to the trained research assistants who will then make contact by telephone to explain the study, answer any questions and obtain informed verbal consent to participate. If a potential participant does not consent to participate in the study, then the research assistant will document the reasons for non-participation and send this information to the research coordinator. The research coordinators in each province will track the numbers of potential participants who are approached to participate in the study, as well as the number of eligible consenters and eligible non-consenters.

All participants who consent to participate will be assigned a unique study identifier to maintain confidentiality. Participants will be randomly assigned (1:1 ratio) to either an intervention or educational control group using a centralized web-based randomization service (RedCaP), a secure, password-protected web application housed by the University of Alberta). All participants will be blinded to group allocation as they will not know what intervention they are receiving. To facilitate this process, two separate consent forms have been created; one for participants allocated to the intervention and another for the educational control group. At the time of the baseline interview, the research assistant will open the electronic files associated with the participants’ study ID and read the appropriate study information and consent form to the participant before proceeding with the collection of demographic data and the baseline survey measures. All participants will receive a copy of the appropriate study information and consent form by email immediately following the baseline interview.

### Control group

Following collection of baseline measures, control group participants will receive an electronic copy of the Alzheimer Society’s [18] *The Progression of Alzheimer’s Disease* by email. This electronic document consists of a five-part series that describes the stages of Alzheimer’s disease. It is written for the person with Alzheimer disease, their family and carers and is freely accessible via the Alzheimer Society of Canada website. Once the data collection is complete, participants in the control group will be given access to MT4C if they wish.

### Intervention: my tools 4 care

Participants randomized to the intervention group will receive sign-in information for the MT4C website, i.e., the website address and a unique username and password to access the site for 3 months; and, (b) an electronic copy of a Toolkit Checklist in which the participant will be asked to document their use of MT4C, i.e., time spent and content accessed, over 3 months. The content of MT4C is summarized in Table 1. All data entered in MT4C by the participants will be kept confidential. Participants allocated to the intervention will also receive an electronic copy of the Alzheimer Society’s [18] educational booklet, *The Progression of Alzheimer’s Disease* after baseline measures have been collected.

**Table 1:** Content of the self-administered on-line transition toolkit

Section	Content
Introduction	The home page is available publicly on the web. (All other pages require logging in.) It contains: • Introduction to the toolkit • Log in box (email/password) • Registration form to create new account (also asks for demographic info) • Password retrieval process • Link to privacy policy, terms of use, etc. • Tutorials and help files
Section 1: About Me	Contains guided activities to help carers to think about and understand transitions. Activities include understanding their inner strengths, what gives them hope, their backup plan in case they are no longer able to provide care, and more. For each activity, users can add formatted text, photos, and attachments such as PDFs.
Section 2: Common Changes to Expect	Contains information about the types of transitions to expect in all areas of their lives, along with quotes from other carers about their experiences (quotes obtained from a previous research study). This section is read-only.
Section 3: Frequently Asked Questions	Contains questions suggested by carers who participated in a past research study, and answers provided by experts and practitioners in the field. This section is read-only.
Section 4: Resources	Contains: • Contact information for provincial and national organizations • Space to add additional contacts • Information on where to obtain other relevant books, brochures, and resources • Links to websites containing relevant, evidence-based information, plus a short paragraph explaining the purpose of each website • PDFs of key brochures and a link to streaming video of “Living with Hope”
Section 5: Important Health Information	Contains sections for entering health information about the person with dementia: • General health information, including allergies and medical history • Names and contact information for doctor, pharmacist, and other healthcare providers • Prescriptions and over-the-counter medications, including dosage, schedule, and reason for each one • Chart for tracking patient’s behaviour over time • Allows users to upload relevant files as PDFs. All information that the caregiver adds to the website is password-protected and will be kept confidential. Only the study administrator will be able to see the information, in the event that the caregiver needs technical assistance with the website.
Additional Features	• Intuitive and easy to use • Ability to change font size for the whole site (user’s preference is saved for future visits) • Ability to print any page or send information to others by email • Mobile site also available so users can log in and see their information. Mobile site is read-only. • Mobile site is cross-platform (will work on any smart phone).

### Measures

#### Caregiver demographic form

Demographic data will be collected from all carers during the baseline interview. These data will include age, gender, marital status, ethnicity, citizenship, education, employment status, occupation, income, religious background, history of chronic health conditions, relationship to the older adult with ADRD, and length of time caring for the older adults with ADRD. Carers will also be asked to report the age, gender, and chronic conditions of the older adult with ADRD and MCC for whom they are providing care.

#### Toolkit checklist

As noted above, participants allocated to use MT4C will be asked to track their use of the intervention i.e. the content accessed and approximate time spent in minutes over the 3 months during which they have access to the intervention. Data collectors will review the Toolkit Checklist with the participants during their scheduled 1 and 3-month telephone interviews. This Toolkit Checklist was used in the pilot study with the Transition Toolkit [16].

#### Qualitative interviews

At 1, 3, and 6 months, semi-structured audio-taped telephone interviews lasting 20–30 min will be conducted with participants allocated to both groups to understand the significant changes they have experienced as carers, as well as what has helped them deal with these changes. For the intervention group participants the interviews also will explore their perceptions of MT4C. For example participants will be asked to describe how MT4C helped them to deal with transitions, what they like most and least about MT4C and about changes that they would make to MT4C.

### Primary outcome measures

The primary outcome variable is the **Mental Component Summary (MCS) score** from the short form Health Survey (SF12.v2) [19]. This outcome was selected because the intervention was designed to primarily address the psychosocial impacts associated with transitions experienced by carers of persons with ADRD [20]. The secondary outcome variable is the Physical Component Summary (PCS) score.

The **SF12.v2** consists of 12 questions that measure functional health and well-being from the client’s perspective, and it has been used in a number of studies involving community-dwelling older adults. The SF12.v2provides scores for eight health domains (physical functioning, role-physical, bodily pain, general health, vitality, social functioning, roles, and emotional and mental health), and two summary, psychometrically-based scores: a physical component summary (PCS) and mental component summary (MCS). The PCS and MCS have a maximum score of 100.The SF-12.v2 has good internal consistency reliability, construct validity, and distinguishes between groups of patients with known clinical differences in physical and mental health, in a variety of client populations [21, 22].

**Fig. 2:**
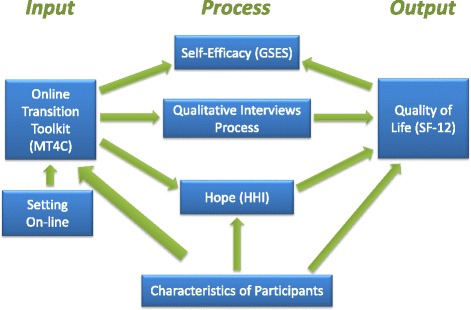
Conceptual framework for MT4C

We hypothesize that hope and general self- efficacy (confidence in the ability to deal with difficult situations) will be the mechanisms through which HRQoL will change over time (see Fig. 2). Hope will be measured by the **Herth Hope Index**, which is a 12-item, 4-point Likert scale that assesses three dimensions of hope: temporality and future, positive readiness and expectancy, and interconnectedness [23]. Scores range from 12 to 48, with higher scores indicating greater hope. The HHI has been found to be reliable and valid measures with family carers [24, 25]. The **General Self-Efficacy Scale** [26] will be used to measure carers’ level of self-efficacy. The GSES is a 10-item scale that is designed to assess one’s ability to deal with adverse situations. Total scores range from 10 to 40, with higher scores indicating greater self-efficacy. The GSES has been reported to be reliable and valid measure across a number of populations [27].

#### Cost of use of health services

The costs of use of all types of health services for all carers will be determined using a version of the Health and Social Services Utilization Inventory (HSSUI) that has been modified for this study. The HSSUI assesses costs from a societal perspective [27] which implies collecting all costs, regardless of who bears them. The broader the perspective taken, the more applicable the study is to social policy decisions.

The initial version of the HSSUI, developed by Browne and colleagues [28], has been tested for reliability and validity [29] as well as tailored to other study populations and used to gather information on service utilization [30]. The HSSUI consists of questions about the respondents’ use of services in eight areas: primary care; emergency department and specialists; hospital days; other health and social professionals; medications; laboratory services; community support services; and other services. It directs participants to reflect back over a specific time period (i.e. 3 months). The HSSUI was also designed to assess direct out-of-pocket expenditures and indirect costs. The cost data will be derived from “quantity” data reported on the HSSUI and “price” data obtained by our team from the Ontario Ministry of Health, Alberta Ministry of Health and Long-Term Care Health Data Branch Web Portal (https://hsim.health.gov.on.ca/HDBPortal). The product of the number of units of service (quantity) and unit cost (price) is total cost.

### Power and sample size

A convenience sample of 180 carers of older adults with Alzheimer’s disease/related dementias and MCC will be recruited to participate in this study; 90 in Ontario, and 90 in Alberta. This sample size (45 per group × 2 groups × 2 provinces) will provide 80% power (alpha = 0.05) to detect a mean difference of 2 increases in the MCS of the SF12v2 (primary outcome) with a standard deviation of 2.5.

### Data collection

Trained, site-specific research assistants (data collectors) will collect all study data during pre-arranged, telephone interviews with consenting study participants at four time points: baseline, 1 month, 3 months and 6 months. All interviews will be digitally recorded and will range from 30 to 60 min in length depending on the participant’s group allocation and the data time point. Table 2 outlines the schedule of data collection by group. Data collectors will reconfirm the participant’s eligibility and obtain their verbal consent to participate prior to each telephone interview.

**Table 2:** Schedule of data collection (Intervention and control groups)

	Initial (Baseline)	1 month (day 28)	3 months	6 months
Quantitative Data:				
Demographics	X			
Health-Related Quality of Life (SF-12v2)	X	X	X	X
Self-Efficacy (GSES)	X	X	X	X
Hope (HHI)	X	X	X	X
Use of Services (HSSUI)	X		X	X
Toolkit Checklist		I	I	
Qualitative Data		I	X	X

X = intervention and control groups; I = intervention group only

During each interview, research assistants will record participants’ responses to the survey questions electronically using e-fillable surveys accessible through ReDCap. The surveys are only marked with the participant’s unique study ID, and accessed on an encrypted study laptop dedicated to the study. Digital recordings of all semi-structured interviews will be labeled with each participant’s study ID and the respective data time point, and then uploaded to the secure SharePoint site. Interview data will be anonymized at the time of transcription, in preparation for analysis.

At the first telephone contact all participants will be sent, electronically, a study brochure with the research coordinator’s contact information, and a copy of all study questionnaires for reference only. At the time of data collection, the interviewer/data collector will obtain the participant’s verbal consent and remind them that they may follow along with the interview using the questionnaires sent previously. Research assistants will track the dates and times of all contact with participants, including email communication.

To promote participant retention, data collectors will send standardized reminder messages, by email, to participants 2 weeks prior to each scheduled interview. In the event that the participant requests a change in the date and time of an interview, or misses a scheduled interview, every effort will be made to re-schedule the interview within a reasonable amount of time, i.e., within 1 week for the 1-month interview, and within 3 weeks for the 3- and 6-month interviews.

### Data analysis

All data will be cleaned and reviewed for potential outliers. Where indicated, data collection forms and interview transcripts will be reviewed to confirm or correct any data queries. The following is the proposed analysis plan for each research question.

#### Question 1: primary outcome analysis

Intent to treat analysis using generalized estimating equations (GEE) was selected as the analytical model. Analyses will be implemented using PROC GENMOD in SAS Version 9.3 [31]. The GEE model will include the following variables: group (intervention, control), time, group x time interaction, and selected covariates (if a significant intervention effect is detected in a model without covariates). If included in the GEE, covariates will include age, gender and other variables where significant group differences are seen in a comparison at baseline. The outcome of interest is a continuous variable SF12v2 MCS. The variable of primary interest is the group and time interaction, which indicates a treatment effect if statistically significant [32]. Time will be modelled based on assessment of a plot of the mean MCS values for both groups at each time period, with time treated continuously if a linear trend is observed and categorically if a non-linear trend is observed.

GEE does not require a normal distribution and SAS offers a number of distributional choices and associated link functions. The distribution (and link) selected will be the one that best matches the distribution of MCS scores for study participants. MCS is negatively skewed in many populations, with few suitable functional distributions for this pattern. If the distribution of MCS scores is significantly negatively skewed, a reflected transformation will be applied to the MCS scores in an effort to normalize them [33] and then the normal distribution and identity link will be applied to the transformed data in the GEE.

### Primary analysis and handling of missing data

GEE assumes that data are missing completely at random [32] an assumption that is difficult to verify [34]. In fact, all missing mechanisms are best thought of as a continuum between missing at random and missing not at random [35], with the current recommendation from experts in the field of missing data being to base the primary analysis on the missing at random assumption [36]. Thus, after the missing data have been multiply imputed, each data set will be analyzed using GEE, and the results from these multiple analyses will be pooled to obtain an overall inference.

### Secondary analysis

Secondary (health-related) outcomes to be explored include: physical functioning as measured by the SF12v2 Physical Component Summary (PCS), HHI and GSES. The analyses relating to all secondary outcomes will be the same as described above for the primary analysis. The distribution and missing patterns for each secondary health-related outcome will be examined to select the appropriate methods for the analytical model (GEE) and for handling missing data.

#### Question 2: qualitative analysis

Content analysis will be used, consistent with a qualitative descriptive approach [37]. Initially, 3–4 research team members will independently read a sample of the transcripts, label them with codes and look for similarities, differences and patterns in the data. Team members will then meet to discuss codes and develop a coding list that can be used for analysis of the remaining transcripts. The team will meet regularly to discuss, compare, corroborate, and revise codes and group them into categories describing the participants’ experience. NVivo 11 software will be used to manage and support analysis of the study data. Several strategies will be used to enhance the rigor of the qualitative analysis. Repeated, semi-structured, individual telephone interviews with carers over a 3 to 6 month period will help to ensure credibility of the data. Investigator triangulation will involve frequent team meetings to review data coding and analysis. Transferability will be supported through the use of field notes and detailed accounts of the research process. Confirmability will be supported through the use of an audit trail of study decisions.

#### Question 3: cost analysis

The cost of services will be determined by calculating the product of the units of service over the 3-month period (i.e., the quantity) by the unit cost (i.e., the price). The costs used will be based on the values indicated in the costing manuals developed for Ontario and Alberta by the Aging, Community and Health Research Unit. The total cost (with and without hospital stay) and for each of the HSSUI categories will be determined. The data will be summarized using frequencies, means, medians, and standard deviations. GEE will also be used to compare the cost data from baseline to 3 months and then 6 months for both the intervention and control groups.

## Discussion

This study will assess the effectiveness of My Tools 4 Care in supporting family carers as well as the cost of this intervention from a societal perspective. Although multiple internet interventions are available for family carers, this is the first to be based on transition theory. Most interventions for family carers of persons with ADRD have been developed based on stress theories and typically focus on providing information and skills with respect to become a better caregiver. MT4C was developed to focus on supporting family carers as they experience multiple transitions. Family carers have been involved at every stage of the development of MT4C and with their assistance it has been continuously evaluated for feasibility and acceptability and to determine if it meets their needs. It is a flexible multicomponent intervention that can be used throughout the caregiver journey and now the online format gives the intervention additional flexibility and portability. It is self-administered and when the study is completed it will be made available to the public for use.

Very few interventions have examined costs from a societal perspective. It was difficult to find a measure of service use so the HSSUI was adapted to carers and their caregiving experience. For example the adaptation of the HSSUI includes costs such as financial services, or lawyers’ fees. The data from the HSSUI over time, will provided important information about caregiving and the use of health and social services.

Using a mixed method design, the qualitative evaluation will provide insight into what is important and what needs to be changed in MT4C before release into the public domain. As well the qualitative data will increase our understanding of the psychosocial processes that occur during the intervention and identify potential mechanisms of the intervention as well as additional outcomes.

There is a great need for effective interventions to support family carers of older persons with ADRD and MCC living in the community. MCC adds complexity to the care provided by family carers and increases the number of transitions they experience. Transitions can significantly impact the HRQOL of family carers [4], so MT4C has the potential to improve the hope, self-efficacy and HRQoL of family carers.

## Abbreviations

ADRD: Alzheimer disease and related dementias
AS: Alzheimer society
CONSORT: Consolidated standards for reporting clinical trials
GEE: General estimating equations
GSES: General self efficacy scale
HHI: Herth hope index
HSSUI: Health and Social Services Utilization Summary
MCC: Multiple chronic conditions
MCS: Mental component summary
MT4C: My tools 4 care
PCS: Physical component summary
SF12v2: Short form 12 version 2
